# Assessing the impact of climate change and human activity on streamflow in a semiarid basin using precipitation and baseflow analysis

**DOI:** 10.1038/s41598-022-13143-y

**Published:** 2022-06-02

**Authors:** Javad Saedi, Mohammad Reza Sharifi, Ali Saremi, Hossein Babazadeh

**Affiliations:** 1 Department of Water Science and Engineering, Science and Research Branch, Islamic Azad University, Tehran, Iran; 2 Department of Hydrology and Water Resources, Faculty of Water and Environmental Engineering, Shahid Chamran University of Ahvaz, Ahvaz, Iran

**Keywords:** Climate sciences, Hydrology

## Abstract

Assessment of streamflow variations under the influence of climate change and human activity is crucial for sustainable water resource management, especially in semiarid areas. In this study, we first used the Hydrograph Separation Program to separate and analyze the base flow index (BFI) that was impacted directly by human activity and precipitation as an important climate factor from 1967 to 2016 in the Dez River Basin. Second, the Mann–Kendall trend test was used to identify trends and change points. Then, the elasticity coefficient method was applied to calculate the impacts of natural factors and anthropogenic activities. The results of the separation methods showed that the sliding interval method produced a better performance. Furthermore; the analyzed trend test at the annual scale showed a significant decreasing trend for runoff as well as increasing trends for the baseflow index in the four of five sub-basins of the Dez River at confidence levels of 95% and 99%, while the average precipitation in these sub-basins was not significant. Additionally, at the seasonal scale in these sub-basins, the average precipitation in winter showed a significant downward trend, while runoff showed a decreasing trend and the BFI index showed increasing trends in winter, spring and summer. The abrupt change point was determined after the change in the BFI index; the runoff was reduced. The maximum change occurred in the sub-basin tireh which after change point from 1977 to 1993,runoff reduced − 1.49% comparison with the base period( from 1967 to 1976) also elasticity estimation was − 0.46,but after change point in Baseflow index from 1994 to 2016 runoff reduced − 55.02% and elasticity estimation was − 0.65. The baseflow index trend and elasticity estimation also indicated that intensive human activities had more significant effects on the Dez Basin's hydrological processes and streamflow variation.

## Introduction

Climate change and anthropogenic activities are two major factors that impact hydrological processes. Climate variability have led to global warming and changing precipitation patterns, while human activities have changed the temporal and spatial distribution of water resources^1–3^. The water resources of the semiarid regions account for less than 2% of the global total^4^. However, these regions support about 20% of the global population^5^. In these water‐limited areas, Human interferences and climate change impact are of increasing apprehension for water resource strategists and managers. Therefore, Understanding the influence of climate variability and human activities will be beneficial for developing sustainable water management strategies. Many studies have used conceptual, physical, black-box numerical models and empirical statistics (for instance, Zhang et al.^6^, Sterling et al.^7^, Vogel et al.^8^, Siriwardena et al.^9^, Chang et al.^10^ and Zhao et al.^11^) and a method based on the water-energy balance (for instance, Ma et al.^12^, Wang et al.^13^, Budyko^14^) assessed the impacts of climate change and human activities on hydrological processes. Streamflow is the most important component of the hydrological cycle and is typically divided into two components for hydrograph separation: base flow and quick flow or storm flow. In arid and semiarid areas, streamflow is mainly supplied by baseflow, making baseflow an important hydrologic characteristic^15,16^. Therefore, understanding the role of base flow in the streamflow processes is critical for the quantifying and identifying direct runoff and groundwater storage^17–20^. The responses of base flow to climate change and anthropogenic activities are different. There is still a lack of research, especially on the characteristics of base flow change and its influencing factors^21^. Human interferences, such as land use and land cover changes, water flow or storage through river diversions, and groundwater pumping, affect the baseflow recession process. Baseflow is difficult to measure directly compared with runoff. Methods used for baseflow separation are limited to hydrograph analysis, which utilizes existing river flow data and provides estimates of baseflow without the need for complex modeling, detailed knowledge of soil characteristics, or costly site investigations^22^. Numerical simulations are currently the most widely used to quantify baseflow because the models are easy to operate and suitable for long-term hydrologic series^23^. Wilby et al.^24^ used a hydrological model to simulate the relationship between climate change and watershed base flow, and the results showed that the base flow was most affected by climate change in wet seasons but was most affected by land use in dry seasons. Mwakalila et al.^25^ used the baseflow index (BFI) method to examine the influence of physical catchment properties on baseflow in these water‐limited environments; they showed that the BFI has a strong relationship with the drainage density index. Fan et al.^26^ applied digital filtering to separate baseflow from streamflow and then to investigate the dynamics of baseflow in four headstreams of the Tarim River Basin. The baseflow appeared to show obvious seasonal variation; the lowest baseflow mainly occurred in winter, and the largest baseflow occurred in summer. Heydari Tasheh Kabood et al.^27^ investigated the effects of climate change on stream flow variation in the Urmia Lake basin as the wettest basin in Iran. Result showed that the Urmia Lake basin face with decreasing stream flows, declining precipitation and rising temperatures in the future furthermore, precipitation, as an important climate factor, plays a vital role in streamflow variation. Novotny and Stefan^28^ showed that variation in precipitation patterns plays a vital role in streamflow trends and sediment in various regions across the United States. Therefore, The objective of this study was to assess the impact of climate change and human activity on streamflow in the Dez River Basin, located in the semiarid area of Iran, by performance baseflow separation methods to analyze the base flow/quick flow characteristics under a long time scale and analysis trend of the base flow index (BFI), which quantifies the contribution of baseflow from streamflow impacted directly by human activity, Also trend of the precipitation as an important climate factor.

## Data and methodology

### Study area

The Dez Basin is one of the largest watersheds in Iran (more than 21,000 km^2^) and is located in the Zagros Mountains in southwestern Iran between 31° 35/51″ and 34° 7/46″ N and 48° 9/15″–50° 18/37″ E in a semiarid area with a Mediterranean climate. The Dez River is the mainstream river in this basin, and Sezar and Bakhtiari are the two main branches of this river. The Sezar River flows in the northernmost part of the Dez Basin and consists of two branches: Marbareh and tireh. The Bakhtiari River is the second main branch of the Dez River, which originates from the eastern heights and slopes of the Oshtorankuh Mountains. Downstream of this basin is Dez Dam, where the storage of the Dez reservoir is important in water supply for extensive agricultural land (125,000 hectares), domestic and industrial sectors, generating electricity, and flood control. According to the downstream water supply situation, it is necessary to analyze the impact of climate change and land use factors to develop and utilize water resources for the basin, which will provide a reference for rational planning.

**Figure 1:**
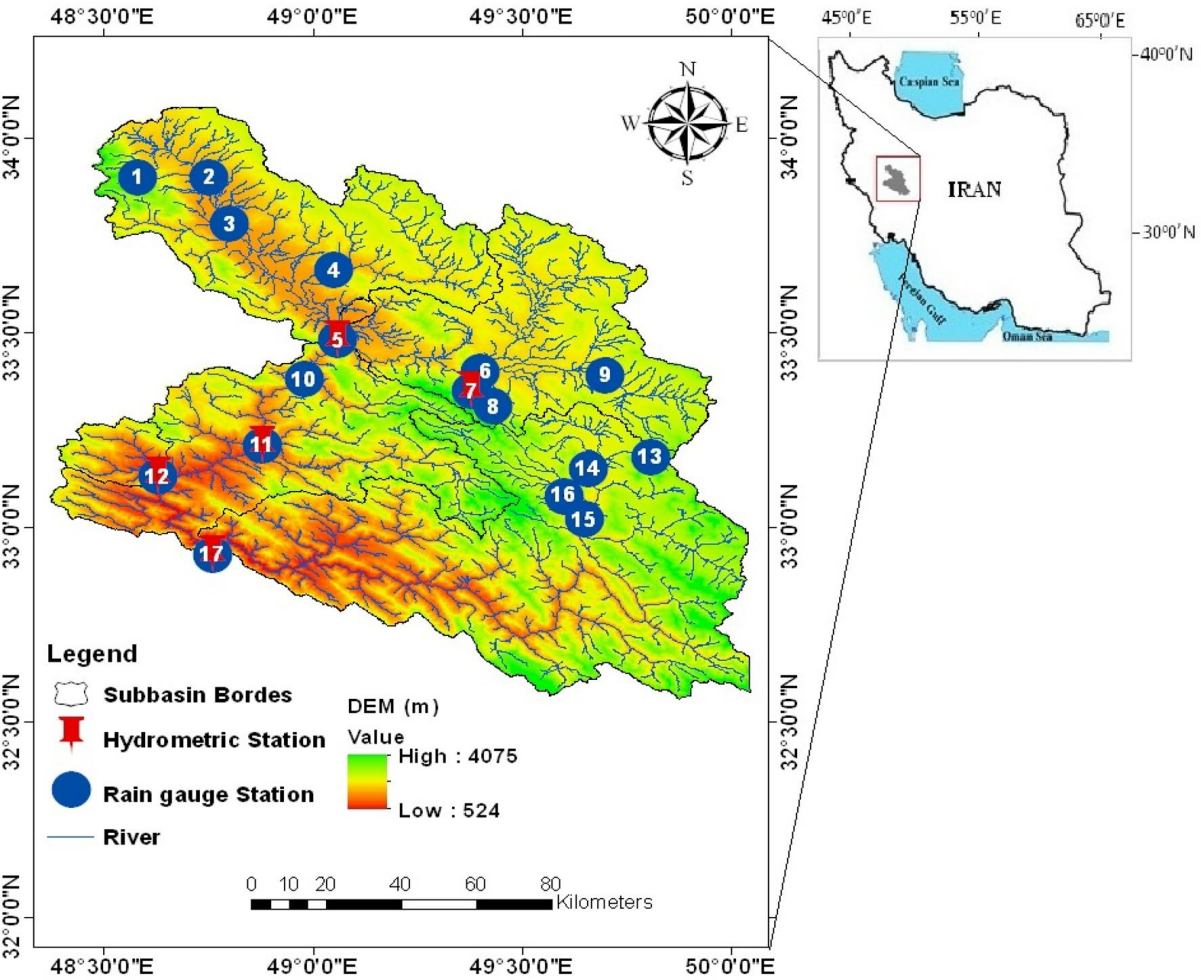
Location, topography, and stations of the Dez River Basin. This figure is created using version 10.2 of ArcGIS software (https://www.arcgis.com).

In Fig. 1 a digital elevation model at a resolution of 30 m, which was downloaded from the U.S. The Geological Survey (USGS) (http://www.usgs.gov) shows the location of the Dez River Basin in Iran, and hydrometric and meteorological stations also illustrate the five sub-basins in this basin. The study area has unevenly distributed precipitation. The temporal and spatial distributions of precipitation in this basin are different. In terms of the temporal distribution of precipitation, the least precipitation occurs in summer due to the high tropical system, and the precipitation is relatively low and sometimes zero. The subtropical high-pressure belt moves to lower latitudes in winter, and the opposite edges pass over this region. These fronts are the source of precipitation that occurs in the basin. In terms of the spatial distribution of precipitation, the average annual precipitation in the northern and eastern parts of the Dez basin is lower than that in other basin areas.

### Data

The Iran Water Resources Management Company acquired daily streamflow and Rain gauging records over 50 years (1967–2016) from five sub-basins of the Dez Basin, summarized in Table 1. The annual period was taken as the hydrological year, and the seasonal time series was divided into four seasons: fall (23 September–21 December), winter (22 December–20 March), spring (21 March–21 June), and summer (22 June–22 September). A digital elevation model at a resolution of 30 m, which was downloaded from the U.S. The Geological Survey (USGS) (http://www.usgs.gov) was used to extract the hydrological model's elevation, and slope and assess the impact of land-use change on the hydrological regime using Land use data extracted from Landsat 4, 5 and 8 images provided by OLI sensors for 1975 and 2015.These images were processed by visual interpretation with supervised classification supported by a geospatial analysis platform in ArcGIS 10.2. (ESRI, USA). In Table 1 type R&H refers to Rain gauging and Hydrometric Station and type R refers to Rain gauging Station.

**Table 1:** Information for the rain gauging and hydrometric stations in the Dez Basin.

Sub basin	River name	Drainage area (km^2^)	No.	Type	Station name	Longitude (°)	Latitude (°)	Elevation (m)
S1	Tireh	3400	1	R	Venaei	48.58	33.90	2000
			2	R	Brojerd	48.75	33.90	1540
			3	R	Rahimabad	48.80	33.78	1490
			4	R	Marok	49.05	33.66	1540
			5	R&H	Dorod	49.06	33.48	1400
S2	Marbreh	2655	6	R	Chamzman	49.40	33.40	1830
			7	R&H	Darehtakht	49.38	33.35	1940
			8	R	Kemandan	49.43	33.31	2080
			9	R	Aligodarz	49.70	33.39	2006
S3	Sezar	3281	10	R	Chamchit	48.98	33.38	1290
			11	R&H	Speiddasht	48.88	33.21	970
S4	Sorkab	336	12	R&H	Kasvar	48.63	33.13	770
S5	Bakhtiyari	6432	13	R	Abbarik	49.81	33.18	2470
			14	R	Kazemabad	49.66	33.15	2000
			15	R	Kakolestan	49.65	33.02	1780
			16	R	Sokaneh	49.60	33.08	1330
			17	R&H	Tangpang	48.76	32.93	600

### Methodology

**Figure 2:**
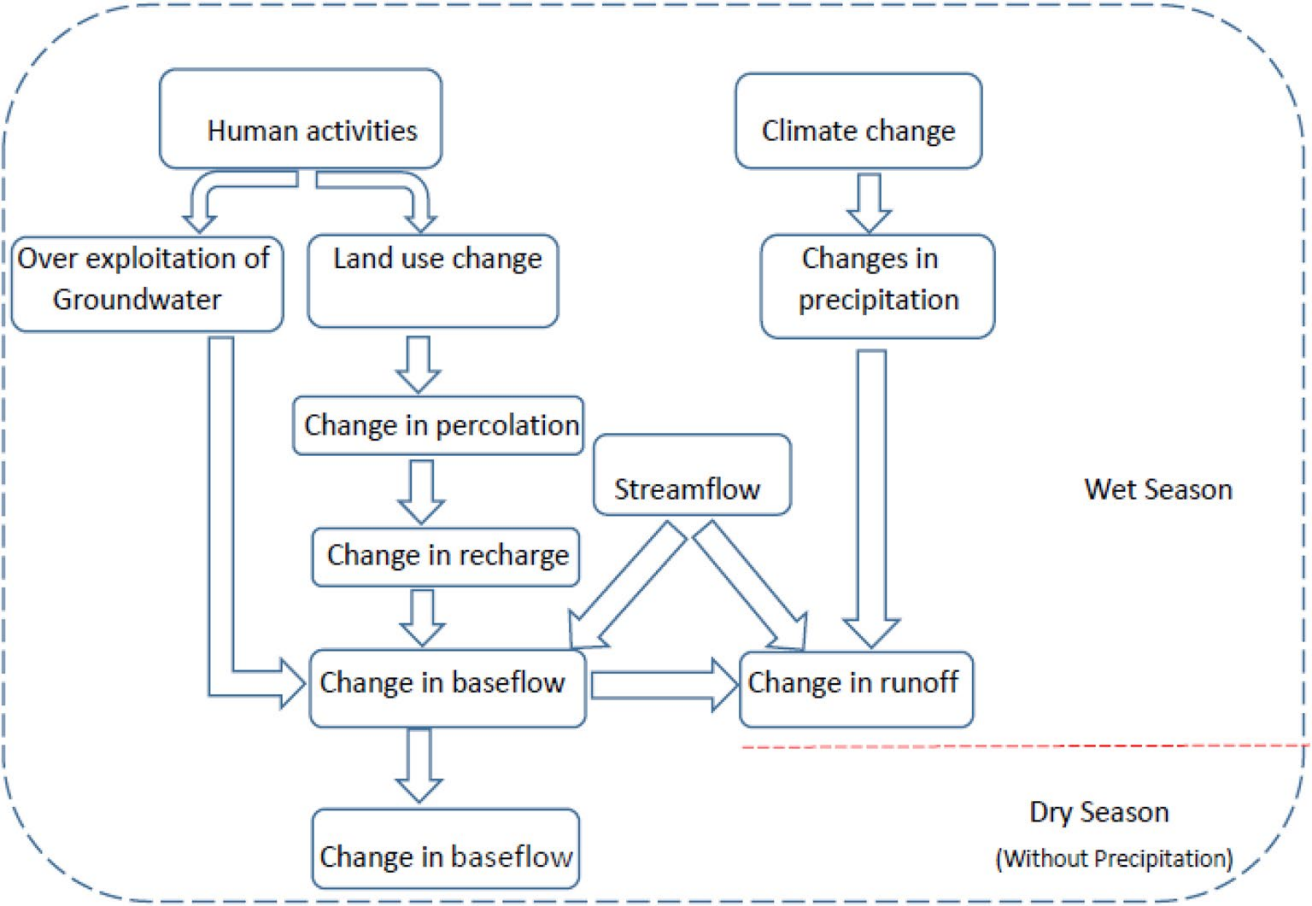
Conceptual relationship between human activity and changing climate impact in the Semiarid Basin.

The analysis contribution of climate and land-use-induced alteration to streamflow follows the water balance for the long-term time in a watershed, and the equation can be expressed as:

1 $$ {\text{If}}\;{\text{Q}} = {\text{q}}_{{\text{s}}} + {\text{q}}_{{\text{b}}} + {\text{q}}_{{\text{n}}} $$

2 $$ {\text{q}}_{{\text{s}}} + {\text{q}}_{{\text{b}}} + {\text{q}}_{{\text{n}}} = {\text{P}} - {\text{E}} + \Delta {\text{S}} $$

where Q is the streamflow, q_s_ is the surface runoff, q_b_ is the groundwater contribution to runoff, which is the definition of baseflow, and q_n_ is the net flux of any water leaving or entering the region other than precipitation (e.g., irrigation, water diversions, and groundwater flux across the basin boundaries).furthermore, P is the mean annual precipitation, E represents the mean annual actual evapotranspiration and DS is the change in stored water within the area. The general assumption that DS is negligible in most cases in the long term. Also, q_n_ in Eq. (2) can be neglected, at least on a regional scale. Because in the some watersheds where hydraulic heads have changed significantly in the past, biased recharge estimates may be generated. Moreover, considering that water transfer projects have been carried out from this basin to the central parts of Iran. Concerning the stated assumptions, Eq. (2) simplifies to (Fig. 2):

3 $$ {\text{q}}_{{\text{s}}} + {\text{q}}_{{\text{b}}} = {\text{P}} - {\text{E}} $$

Precipitation also baseflow and runoff, which are the main components of streamflow, can be obtained from actual observations. The closest neighbor station has been used to estimate a target station to fill gaps in missing data^29^, the Thiessen polygon method^30^ in ArcGIS 10.2 software was used to calculate the average precipitation in each subbasin.

#### Analysis variation of baseflow using BFI index

The baseflow index (BFI) was introduced by the Institute of Hydrology^31^, and this index is used as an effective parameter in modeling runoff. The BFI is then obtained by dividing the base flow rate by the total flow volume for each year or the entire period^32,33^, expressed as a fraction or percentage as presented in equation:

4 $$(\text{BFI})\text{ Index}=\frac{\text{Baseflow volume}}{\text{Total flow volume}}$$

It is determined after the hydrograph separation has been completed. Methods for separation generally divide the flow into one quick and one delayed component using automated time-based separation. This research uses Hydro Office 2015 (BFI + 3.0)^34^ for separation Base flow is free to download (http://hydrooffice.org). The main baseflow separation methods are provided in the following sections.

##### HYSEP method

The HYSEP method was developed by Sloto and Crouse to separate the base flow and surface runoff components of daily streamflow^35^. Methods of HYSEP is computed using equation:

5 $$ \text{N}={\text{A}}^{0.2}$$

where the variable N is the number of days after which surface runoff ceases and A is the drainage area in square miles^36^. The HYSEP method includes three approaches. Fixed interval method (FIM) this method assigns the lowest discharge in each interval (N) to all days in that interval starting with the first day of the period of record, also sliding interval method (SIM) this method finds the lowest discharge in one half the interval minus 1 day [0.5(2 N* − 1) days] before and after the day being considered and assigns it to that day, and local minimum method (LMM) this method checks each day to determine if it is the lowest discharge in one half the interval minus 1 day [0.5(2 N* − 1) days] before and after the day being considered.

##### BFLOW method

The Lyne and Hollick algorithm (LHA) was first suggested by Lyne and Hollick in the frequency spectrum of a hydrograph^37^. Long waves will be more likely to be associated with the base flow, while the high-frequency variability of the streamflow will primarily be caused by direct runoff. It should, therefore, be possible to identify the base flow by low pass filtering the hydrograph. Nathan and McMahan^38^, the most acceptable results occurred in this method when the filter parameter was within the range of 0.90–0.95 with an average value of 0.925. Additionally, Smakhtin and Watkins^39^ found that the optimal filter parameter values normally fluctuated between 0.985 and 0.995, and a value of 0.995 was recommended for the separation of the daily base flow. This is represented by equation:

6 $${q}_{f(i)}=\propto {q}_{f\left(i-1\right)}+\left({q}_{\left(i\right)}-{q}_{\left(i-1\right)}\right)1+\propto /2$$

Subject to ${q}_{f(i)}\ge 0,{q}_{f(i)}$ direct runoff filtered at time step ${q}_{f(i-1)}$ direct runoff filter at time i-1,$\propto$ filtering parameter, ${q}_{(i)}$ The total flow in time i,${q}_{(i-1)}$ total flow at time step i − 1 and q_b_ = q − q_f_ is the base flow.

##### Calibration of baseflow separation methods

The coefficient of determination (R^2^) value used as an objective function in mathematical programming formulation for calibration is calculated using equation:

7 $${\text{R}}^{2} =\frac{(\sum {\left({\text{Q}}_{0}-{\overline{\text{Q}} }_{0})\left({\text{Q}}_{\text{S}}-{\overline{\text{Q}} }_{\text{S}}\right)\right)}^{2}}{{{\sum \left({\text{Q}}_{0}-{\overline{\text{Q}} }_{0}\right)}^{2}\sum \left({\text{Q}}_{\text{S}}-{\overline{\text{Q}} }_{\text{S}}\right)}^{2}}$$

where Q_o_ and Q_s_ are the observed streamflow in the dry season and the simulated base flow, respectively; Qo and Qs are the average observed streamflow in the dry season and the simulated base flow, respectively.

#### Statistical analyses

##### Mann–Kendall trend test and change points

Mann^40^–Kendall^41^ is a nonparametric method that many researchers in meteorology and hydrology have widely utilized to analyze the time-series change trends of runoff, temperature, and precipitation, which are usually nonnormally distributed. The mathematical equations for calculating Mann–Kendall Statistics *S* and standardized test statistics *Z* are as follows:

8 $$S=\sum_{i=1}^{n-1}\sum_{j=i+1}^{n}sign({x}_{j}-{x}_{i})$$

9 $$ S\left( {x_{j} - x_{i} } \right) = \left\{ {\begin{array}{*{20}l} { + 1} \hfill & {if\;\left( {x_{j} - x_{i} } \right) > 0} \hfill \\ 0 \hfill & {if\;\left( {x_{j} - x_{i} } \right) = 0} \hfill \\ { - 1} \hfill & {if\;\left( {x_{j} - x_{i} } \right) < 0} \hfill \\ \end{array} } \right. $$

10 $$Var(S)=\frac{\left[n(n-1)(2n+5)\right]}{18}$$

11 $$ Z = \left\{ {\begin{array}{*{20}l} {\left( {S - 1} \right)/\sqrt {Var} \left( S \right)} \hfill & {if\;S > 0} \hfill \\ 0 \hfill & {if\; S = 0} \hfill \\ {\left( {S + 1} \right)/\sqrt {Var} \left( S \right)} \hfill & {if\;S < 0} \hfill \\ \end{array} } \right. $$

where x_1_, x_2_, x_3_, …, x_n_ are the data arranged in time, and n represents the amount of data. The null hypothesis is accepted if $\left|\text{Z}\right|\le {\text{Z}}_{(1-\propto /2)}$ at the $\propto$ the level of significance; thus, the trend is not significant. The positive and negative values of Z represent upward and downward trends, respectively. When the absolute value of Z is greater than or equal to 2.58, 1.96, and 1.28, it is adopted by the 99%, 95%, and 90% confidence levels, respectively. The software utilized for this purpose was TREND, a product from the Cooperative Research Centre (CRC) for Catchment Hydrology’s (CRCCH) Climate Variability Program^42^. For detecting changing points by using the M–K test, S_K_ is defined as a variable, as follows:

12 $$ S_{K} = \mathop \sum \limits_{i = 1}^{K} r_{i} \quad {\text{k}} = 2,3 \ldots ,{\text{n}} $$

13 $$ S_{i} = \left\{ {\begin{array}{*{20}l} 1 \hfill & {x_{j } < x_{i} } \hfill \\ 0 \hfill & {x_{j} \le x_{i} } \hfill \\ \end{array} } \right\}\quad {\text{j}} = 1, \ldots {\text{i}} $$

where X_i_ and X_j_ are the hydrologic variables when the time series data are i and j, and S_k_ is the count for the X series when X_i_ is larger than X_j_. Subsequently, UF_k_ is used to evaluate the positive sequence of the variable X:

14 $$ {\text{UF}}_{{\text{K}}} = \frac{{{\text{S}}_{{\text{k}}} - {\text{E}}\left( {{\text{S}}_{{\text{k}}} } \right)}}{{\sqrt {{\text{Var}}({\text{S}}_{{\text{k}}} )} }}\quad {\text{k}} = 1, \ldots ,{\text{n}} $$

In the equation, UF_K_is is the standard normal distribution; E (S_k_) and Var(S_k_) represent the mean and variance of S_k_, respectively. At a given significance level, if the UF_k_ value was more significant than 0, it indicated that the series presented a rising trend; otherwise, it presented a declining trend.

#### Elasticity estimation

The climate elasticity of runoff may be defined as the proportional change in runoff, *R*, to the change in a climatic variable such as precipitation. Sankarasubramanian et al.^43^ introduced the nonparametric estimator:

15 $${\varepsilon }_{P}=\text{median}\left(\frac{{\text{R}}_{\text{i}- }\overline{\text{R}}}{{\text{P} }_{\text{i}-}\overline{\text{P}} }\frac{\overline{\text{P}} }{\overline{\text{R}} }\right)$$

where *R*_i_ and *P*_i_ are the runoff depth and precipitation (mm) of the *i*th year, respectively; *R* and *P* are the mean value of the runoff depth and precipitation (mm); ε_P_ is the precipitation elasticity of runoff, and ε_*P*_ reflects the impacts of natural factors and anthropogenic activities since the catchments are heavily regulated via processes such as water diversions or control dams^44^.

## Results

### Analysis of precipitation variation

Summary statistics of the annual and seasonal averages of precipitation over 50 years (1967–2016) were calculated for every subbasin, as shown in Table 2. On an annual basis, precipitation is spatially uneven upstream and the Dez River downstream. The maximum value of mean precipitation is obtained at subbasin S4 (955.2 mm) downstream of the Dez River, and the minimum value of mean precipitation is obtained at subbasin S1 (484.3 mm) upstream of the Dez River. The minimum coefficient of variation (C_V_) was 0.20 (subbasin S1), and the maximum variation was 0.28 (subbasin S4). This indicates that interannual variation is more stable in subbasin S1 than in subbasin S4. On the seasonal basis, most precipitation in every subbasin occurs during the rainy season in winter, fall, and spring. The minimum and maximum mean values of precipitation in the winter season were 201.9 mm (subbasin S1) and 464.4 mm (subbasin S4), respectively, and those in the fall season were 147.7 mm (subbasin S1) and 292.4 mm (subbasin S4), respectively. The spring seasons were 133.6 mm (subbasin S1) and 198.6 mm (subbasin S5), respectively, but the precipitation variability (C_V_) in winter was relatively smaller than that in fall and spring in all sub-basins. The minimum and maximum C_V_ ranges in the winter season were 0.34 (subbasin S1) and 0.40 (subbasin S2), respectively, in the fall season were 0.40 (subbasin S1) and 0.56 (sub-basins S3 and S4). In the spring season, they were 0.40 (subbasin S1) and 0.56 (subbasin S4), respectively, but summer, as a dry season (22 June–22 September), had the lowest rainy day with insignificant precipitation. The minimum average precipitation was 1.1 (sub-basins S1, S3, S4) mm, and the maximum average was 1.9 mm (subbasin S5), which means that this amount of precipitation cannot affect the flow of the river.

**Table 2:** Statistical characteristics of annual and seasonal precipitation.

Sub-basin	Index	Wet season			Dry season	Annual
		Fall	Winter	Spring	Summer	
S1	Max	312.9	373.4	229.1	–	712.3
	Min	61.7	74.8	33.7	–	258.5
	Avg	147.7	201.9	133.6	1.1	484.3
	CV	0.40	0.34	0.40	–	0.20
S2	Max	323.9	449.3	272.1	–	807.1
	Min	47.1	82.6	32.1	–	275.4
	Avg	152.5	215.6	142.7	1.3	512.5
	CV	0.41	0.40	0.42	–	0.24
S3	Max	702.5	609.5	354.5	–	1130.2
	Min	28.5	141.1	21.5	–	417.1
	Avg	217.7	337.8	155.3	1.1	711.7
	CV	0.56	0.36	0.49	–	0.25
S4	Max	807.5	942	513.5	–	1543
	Min	39.5	162	32.2	–	493.5
	Avg	292.4	464.4	188.3	1.1	955.2
	CV	0.56	0.37	0.56	–	0.28
S5	Max	636.8	705.9	461.8	–	1286.9
	Min	62.3	143.9	57.8	–	426.6
	Avg	237.7	322.6	198.6	1.9	755.6
	CV	0.44	0.39	0.41	–	0.26

### Analysis of streamflow variation using baseflow separation methods

The quantity of runoff depends on variation base flow that is spatially and temporally influenced by several factors, including geology, topography, climatic season, and anthropogenic activities^45^. Therefore, the separation method was used to analyze the baseflow and runoff variation to identify the quantity of variation streamflow in this study; therefore, the average daily streamflow variation over 50 years (1967–2016) during the wet and dry seasons were analyzed, and the baseflow separation method is provided in Table 3. The volume of streamflow in the total subbasin in the dry season is less than that in the wet season because in the dry season, no precipitation occurs, and groundwater and snow melts are the primary sources of streamflow. Therefore, daily streamflow records during the dry season were used to calibrate models and separate the daily streamflow's base flow and surface runoff components in every subbasin. During the dry season, the streamflow volume in every subbasin is different. Subbase S5 showed the highest average volume of streamflow (77.5 m^3^ s) during the dry season compared with the other sub-basins because the drainage area of this subbasin is more than that of the other sub-basins, a large part of the subbasin is mountainous and snow-covered, and snow melts are a source of this streamflow. In contrast, the lowest average volume of streamflow was found in subbasin S4 (1.1 m^3^ s) because the drainage area of this subbasin is smaller than that of the other sub-basins. On the other hand, in subbasin S3, the average volume of streamflow (15.1 m^3^ s) is greater than that in sub-basins S1 (2.5 m^3^ s) and S2 (2.9 m^3^ s) because the drainage area of this subbasin is greater than that of sub-basins S1 and S2. As a result, the base flow index (BFI) and the coefficient of determination (R^2^) value were used to compare the analysis results of baseflow separation methods in every subbasin. The BFI index is a relative measure with no units, and a BFI close to 1.0 means that a river has a high proportion of baseflow^46^. Based on the results, five sub-basins showed the highest BFI average values and highest correlation coefficient (though calibrated) computed by the sliding interval method (SIM). In each of the sub-basins over the dry season, the estimated BFI was close to 1.0.

**Table 3:** Average Streamflow over the wet and dry seasons and outcome of baseflow separation.

Sub-basin	Variable (unit)	Wet season	Dry season		Outcome methods			
					FIM	SIM	LMM	LHA
S1	Streamflow (m^3^)	18.9	2.5	Base flow	2.3	2.4	2.2	2.2
				BFI index	0.94	0.97	0.91	0.94
				R^2^	0.93	0.94	0.89	0.84
S2	Streamflow (m^3^)	8.0	2.9	Base flow	2.7	2.8	2.6	2.7
				BFI index	0.95	0.97	0.93	0.95
				R^2^	0.91	0.94	0.81	0.47
S3	Streamflow (m^3^)	52.03	15.1	Base flow	14.3	14.6	14.1	13.8
				BFI index	0.97	0.98	0.96	0.95
				R^2^	0.86	0.89	0.85	0.82
S4	Streamflow (m^3^)	7.6	1.1	Base flow	1.6	1.9	1.2	1.3
				BFI index	0.96	0.98	0.93	0.95
				R^2^	0.86	0.93	0.80	0.76
S5	Streamflow (m^3^)	159.10	77.5	Base flow	73.8	75.4	70.7	70.6
				BFI index	0.96	0.98	0.94	0.95
				R^2^	0.91	0.99	0.80	0.78

Therefore, in the sliding interval method (SIM), the average BFI was 0.97 and had the strongest correlation (0.94) compared to the other methods. In subbasin S2 in the sliding interval method (SIM), the average BFI and R2 were 0.97 and 0.94, respectively; in subbasin S3, the BFI and R^2^ were 0.98 and 0.89, respectively; in subbasin S4, the BFI and R^2^ were 0.98 and 0.93, respectively; and in subbasin S5, the BFI and R^2^ were 0.98 and 0.99, respectively.

**Figure 3:**
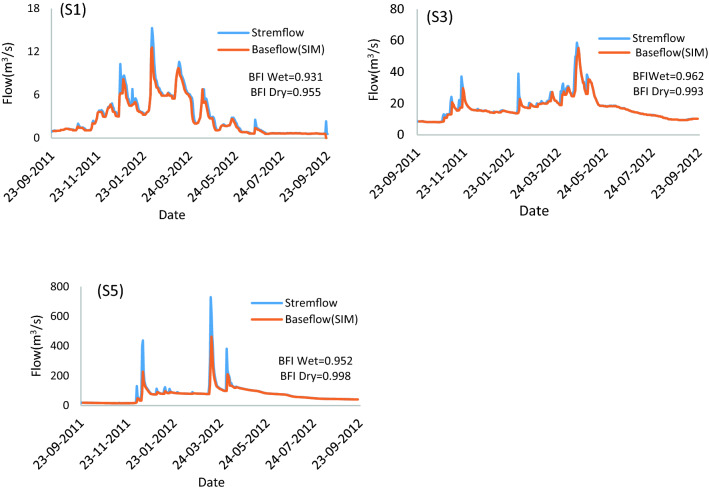
Example of the hydrograph separation results for sub-basins S1, S3, and S5 of the Dez River.

Moreover, as shown in Fig. 3. During the dry season, the contribution of baseflow to streamflow was high, and all the methods except the sliding interval method (SIM) underestimated the baseflow during this period. Based on these results, the analysis of seasonal and annual variation components of streamflow in five sub-basins over 50 years provided in Table 4 found that seasonal patterns of base flow and runoff variation were dissimilar for all sub-basins. In subbasin S1, the baseflow value contribution to streamflow (BFI index) was highest during the summer season (0.97), and the lowest contribution was during the spring season (0.92). Additionally, the BFI fluctuation (C_V_) in the summer season (0.07) was smaller than that in another season because in the dry season, no rainfall occurred, and the flow was relatively steady.During the fall season, the first season of the wet seasons and after the dry season, most of the precipitation infiltrated the soil and charged the groundwater; as a result, the BFI index value was high (0.95), and runoff was low (0.91 m^3^ s) under the influence of this dynamic situation of the subbasin. Fluctuation runoff was much larger (C_V_ = 1.71), while precipitation fluctuation was relatively smaller (C_V_ = 0.40). After this season, high precipitation and soil saturation were observed during the winter season, the BFI index decreased (0.94), and the runoff volume increased (2.5 m^3^ s). The cause of this dynamic variation over this season also fluctuation runoff (C_V_ = 1.06) was much larger than precipitation fluctuation (C_V_ = 0.34).and during the spring season as last of the wet seasons that mean value of precipitation was less than fall and winter, Runoff volume reduces (2.4 m^3^ s) in comparison with winter season but was higher than fall season and BFI index reduce (0.92) because after 2 wet seasons (fall to winter) soil moisture increases and infiltration decreases. On an annual basis, minimum and maximum mean annual runoff is obtained in subbasin S2 (0.65 m^3^ s) and subbasin S5 (16.4 m^3^ s), and minimum and maximum mean annual BFI are obtained in subbasin S4 (0.92) and sub-basins S2 and S3 (0.96), respectively. Moreover, the fluctuation runoff (C_V_) in subbasin S4 (1.08) and the fluctuation BFI (C_V_) in this basin were much larger than those in other sub-basins, which indicates that the interannual variation in this subbasin was unstable.

**Table 4:** Summary statistics characteristics of seasonal and annual separation streamflow.

Sub-basin	Index	Wet season						Dry season	Annual	
		Fall		Winter		Spring		Summer		
		BFI	Runoff (m^3^)	BFI	Runoff (m^3^)	BFI	Runoff (m^3^)	BFI	BFI	Runoff (m^3^)
S1	Max	0.98	9.4	0.98	15.1	0.96	12.3	1.00	0.98	7.97
	Min	0.91	0.13	0.89	0.21	0.86	0.17	0.87	0.90	0.27
	Avg	0.95	0.91	0.94	2.5	0.92	2.4	0.97	0.95	2.01
	Cv	0.09	1.71	0.09	1.06	0.12	1.0	0.07	0.10	0.74
S2	Max	0.99	3.4	0.98	6.3	0.97	4.72	0.99	0.98	3.07
	Min	0.90	0.03	0.91	0.07	0.91	0.15	0.93	0.92	0.10
	Avg	0.96	0.32	0.95	0.82	0.94	0.85	0.98	0.96	0.65
	Cv	0.11	1.81	0.11	1.54	0.13	1.13	0.05	0.10	0.99
S3	Max	0.99	16.4	0.98	43.4	0.97	22.8	0.99	0.98	18.9
	Min	0.91	0.19	0.88	0.80	0.91	0.70	0.96	0.95	0.8
	Avg	0.95	2.2	0.94	6.8	0.95	5.6	0.99	0.96	4.8
	Cv	0.10	1.18	0.11	1.08	0.10	0.90	0.03	0.09	0.71
S4	Max	0.98	6.11	0.98	9.8	0.98	8.9	0.99	0.98	4.9
	Min	0.76	0.03	0.80	0.06	0.87	0.03	0.94	0.91	0.20
	Avg	0.90	1.35	0.87	2.7	0.94	1.12	0.98	0.92	1.70
	Cv	0.21	1.05	0.22	0.70	0.14	1.33	0.05	0.17	1.08
S5	Max	0.99	34.5	0.96	80.8	0.98	45.2	0.99	0.97	39.4
	Min	0.89	0.45	0.86	6.9	0.93	2.5	0.97	0.93	6.2
	Avg	0.94	10.3	0.92	24.1	0.95	14.4	0.99	0.95	16.4
	Cv	0.14	0.77	0.15	0.69	0.12	0.65	0.03	0.12	0.46

**Figure 4:**
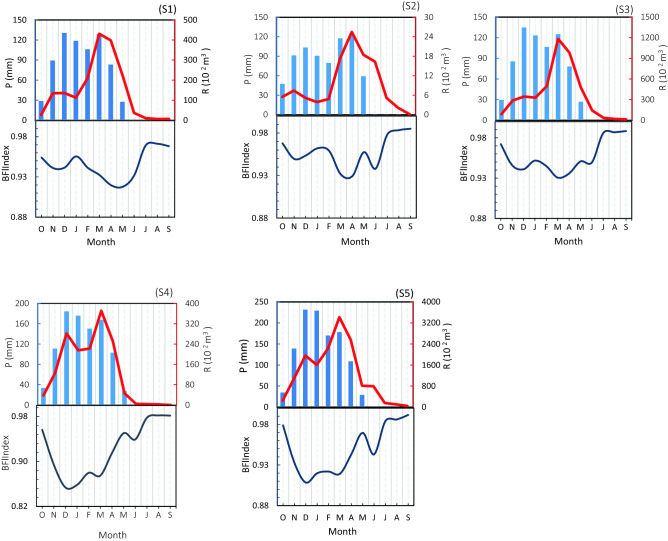
The monthly distribution of precipitation, runoff, and baseflow index in the sub-basins of the Dez River.

The interannual monthly average variable characteristics of precipitation, runoff, and the BFI index from 1967 to 2016 are presented in Fig. 4. On the total subbasin. Although the mean precipitation was high from October to December while runoff was low, the BFI index in these months was high, indicating that most of the precipitation infiltrated the soil and charged the groundwater. The contrast peak of runoff was in March, where precipitation in this month was less than in October to December because the precipitation of previous months, soil moisture, low infiltration, and snow melting in spring caused the rise of runoff. In sub-basins S4 and S5 in December, the cause of high precipitation also occurred, and the peak runoff occurred, which was lower than the peak in March. From June to September, no rainfall occurred, and the BFI was steady and higher than that in other months.

### Trend test and change point analysis

The Mann–Kendall test was used to identify significant trends in precipitation, the BFI index, and runoff in this study. Detecting in the baseflow index (BFI) trends can help us understand the possible links between hydrological processes, anthropogenic activities, and environmental changes^46^. The results of annual and seasonal datasets for 1967–2016 are presented in Table 5. The critical values of statistical test Z$\propto$_/2_ =  ± 1.28, ± 1.96, and ± 2.58; *+** and *** denote the existence of a significant trend at confidence levels of 90%, 95%, and 99%, respectively. According to the trend test, at the annual scale, in subbasin S1, precipitation showed no significant trend; in contrast, runoff showed a downward trend, and the BFI index showed a significant upward trend at a high (% 99) confidence level. This means that anthropogenic activity in this subbasin was greater than climate change. In subbasin S2, runoff also showed a significant downward trend at the high (% 99) confidence level, while precipitation showed a significant downward trend at the low (% 90) confidence level. Additionally, in subbasin S3, precipitation showed no significant trend; in contrast, runoff showed a significant downward trend, and the BFI index showed a significant upward trend at the 95% confidence level. In subbasin S4, the BFI index showed a significant upward trend at a high (% 99) confidence level without a significant trend in runoff and precipitation. In subbasin S5, precipitation showed a significant downward trend at the 95% confidence level, while the BFI index showed a significant upward trend at the 95% confidence level. For seasonal scale. Precipitation showed no significant trend in the fall and spring seasons; only over the winter season in all sub-basins showed a significant downward trend in precipitation, while runoff in sub-basins S1, S2, S3 in the winter and spring seasons also showed a significant downward trend in subbasin S1 in all wet season (fall to spring) runoff showed a significant downward trend. Mahmoodi et al. ^47^ reported that the temporal trend in groundwater levels was monitored at 11 piezometric wells in the Azna-Aligudarz Plain and 13 piezometers in the Doroud-Borujerd, which mainly showed declining trends in groundwater levels.

**Table 5:** Values of *Z* for precipitation, runoff, and the BFI index in the Dez sub-basins.

Sub-basin	Variable (unit)	Wet season			Dry season	Annual	Change point (year)
		Fall	Winter	Spring	Summer		
S1	Runoff (m^3^)	− 2.49**	− 2.97***	− 2.64***	–	− 3.60***	1976
	Precipitation (mm)	0.41	− 2.51**	0.53	–	0.08	–
	BFI index (–)	0.30	1.89*	2.01**	1.76*	2.83***	1993
S2	Runoff (m^3^)	− 1.15	− 2.68***	− 2.43**	–	− 2.63***	1988
	Precipitation (mm)	− 0.18	− 2.59***	− 0.69	–	− 1.94*	–
	BFI index (–)	− 1.16	1.14	0.96	0.68	0.37	–
S3	Runoff (m^3^)	− 0.48	− 2.41**	− 2.51**	–	− 2.14**	1988
	Precipitation (mm)	− 1.02	− 1.84*	− 0.36	–	− 0.77	–
	BFI index (–)	− 0.68	1.66*	2.50**	2.50**	2.20**	1988
S4	Runoff (m^3^)	0.12	− 2.12**	− 0.94	–	− 1.18	–
	Precipitation (mm)	0.44	− 1.95*	0.45	–	0.94	–
	BFI index (–)	1.81*	1.22	1.12	4.16***	2.40**	1990
S5	Runoff (m^3^)	− 0.41	0.24	− 1.39*	–	− 0.57	
	Precipitation (mm)	− 0.78	− 1.67*	− 1.84*	–	− 2.09**	1979
	BFI index (–)	1.09	2.01**	0.47	0.94	2.55**	1989

**Figure 5:**
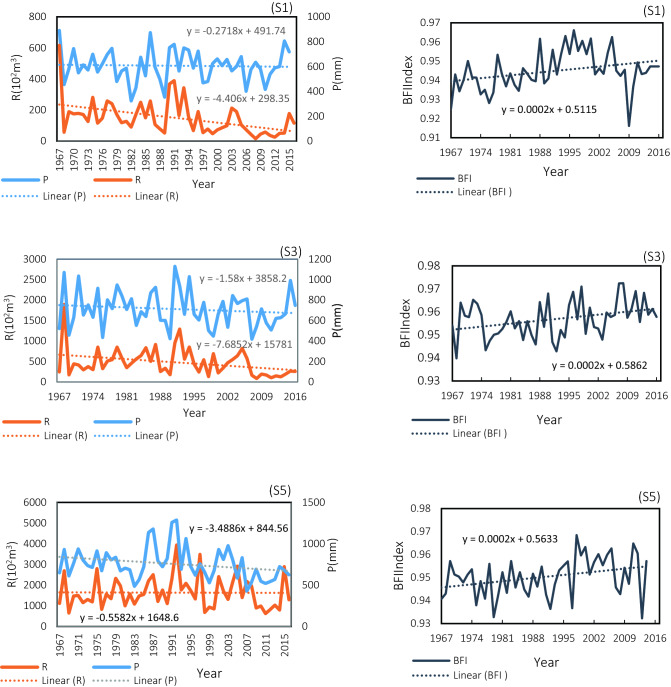
The trend of annual precipitation, Baseflow index, and runoff in the subbasins S1, S3, and S5 of the Dez River.

This finding is in agreement with the results of a study conducted by Noruozi ^48^ Indicated the flow of the Dez Basin in recent decades shows significant monotonic and abrupt changes, which mostly decrease the potential runoff of the basin. Moreover, the BFI index showed a significant upward trend in fall to summer. In subbasin S4, runoff showed a significant downward trend only in the winter, and the BFI index in this subbasin showed a significant upward trend in the fall and summer seasons. In subbasin S5, only the BFI in winter was significant. The annual precipitation trends, BFI, and runoff from 1967 to 2016 are illustrated in Fig. 5.

**Figure 6:**
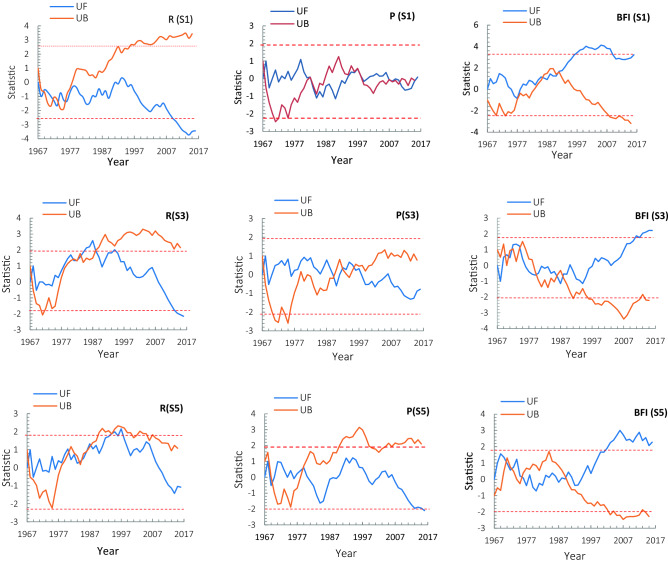
M–K test analysis change point of the (P) precipitation; (R) runoff; (BFI) baseflow index in the subbasins S1, S3, and S5 of the Dez River.

In the Fig. 6. As observed, the precipitation is almost within the limit of − 1.96, which indicates that the precipitation change is stable, and no obvious abrupt point is observed in the precipitation series. In contrast, we detected the change point of runoff and the BFI index over the study period from 1967 to 2016. No obvious abrupt point is observed in the precipitation series in subbasin S1, but the change point for runoff in this subbasin occurred in 1976, and the change point for the BFIindex occurred in 1993, which indicates the impact of anthropogenic activities in this subbasin. On the other hand, the time interval between two change points (runoff and BFIindex) shows the response of surface runoff (rapid) and groundwater (gradual) to anthropogenic activities. In subbasin S2, change point for runoff and precipitation occurred in 1988, which shows the impact of climate change (reduce precipitation) on runoff in this subbasin, in subbasin S3 also shows the change point in runoff and BFI index in 1988 downstream of subbasinS1and S2 without an abrupt point is observed in the precipitation, in the subbasin, S4 change point for BFIindex occurred in 1990, and in subbasin S5 change point for precipitation and BFIindex occurred in 1979 and 1989, respectively.

### Land use change analysis

One of the most important signs of human activity is land use change. In 1967, the Dez River Basin catchment was dominated by rangeland, bare land, and farmland, and rangeland was mostly found in sub-basins S2 (76.72%) and S3 (79.50%). Bare land cover is mostly found in sub-basins S4 (25.29%) and S5 (38.38%). Farming land accounted for mostly 7.30% and 5.94% and was mainly found in the northern part of the basin (sub-basins S1 and S2). Comparing the land-use maps of 1967 and 2016, the most obvious changes occurred in the rangeland areas in sub-basins S1, S2, and S3, which decreased by – 26.44%, − 28.88%, and − 24.63%, respectively (Table 6). In these sub-basins, farming land and bare land also increased from 1967 to 2016; in subbasin S1, farming land and bare land increased by 7.12 and 19.32, respectively. In subbasin S2, farming land and bare land increased by 14.91% (more than other sub-basins) and 13.97%, respectively, and in subbasin S3, farming land and bare land increased by 1.35 and 23.28% (more than other sub-basins), respectively. This shows that those reductions in rangeland areas and increasing farming land and bare land in these sub-basins have been due to population growth and urban development. In the subbasin, the S4 and S5 situations were different as the result of runoff and BFI index trend tests in these sub-basins; bare land was reduced, and rangeland and farmland increased.

**Table 6:** Percentage of land-use changes for sub-basins from 1967 to 2015.

Sub-basin	Period	Land-use classes		
		Farm land	Range land	Bare land
S1	1967	7.30	72.29	20.41
	2015	14.41	45.85	39.73
	Change (%)	7.12	− 26.44	19.32
S2	1967	5.94	76.72	17.34
	2015	20.85	47.84	31.30
	Change (%)	14.91	− 28.88	13.97
S3	1967	1.68	79.50	18.82
	2015	3.03	54.87	42.10
	Change (%)	1.35	− 24.63	23.28
S4	1967	0.52	74.19	25.29
	2015	2.29	79.76	17.95
	Change (%)	1.77	5.57	− 7.34
S5	1967	0.46	61.16	38.38
	2015	3.69	70.48	25.83
	Change (%)	3.23	9.32	− 12.55

**Figure 7:**
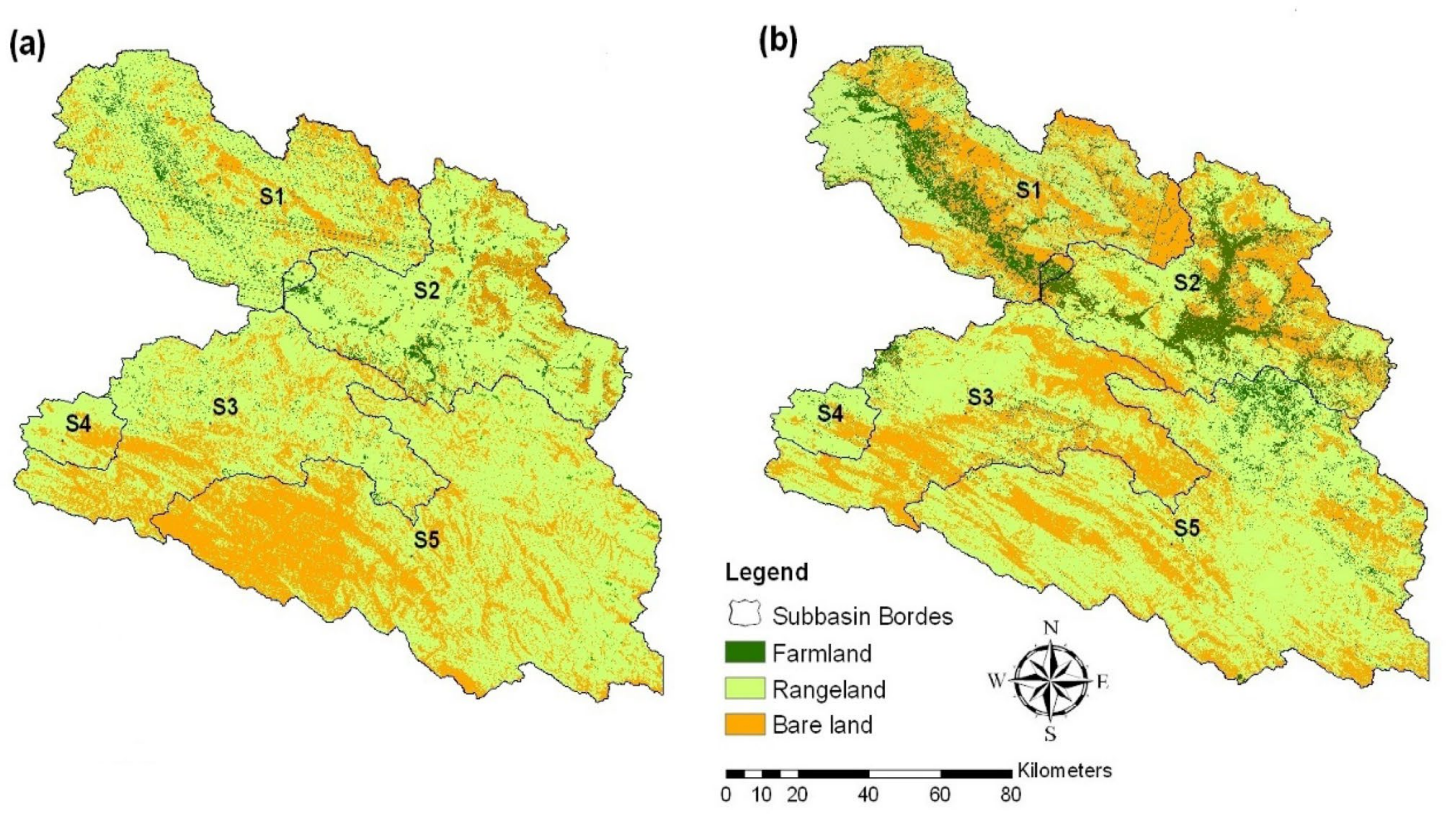
Land-use map of the study area in 1967 (**a**) and 2016 (**b**). This figure is created using version 10.2 of ArcGIS software (https://www.arcgis.com).

The spatial distribution of the major land-use classes in 1967 and 2016 is shown in Fig. 7. The available could-free Landsat 5 and 8 images provided by OLI sensors that cover the whole basin in a mosaic manner within the same season which was downloaded from the U.S. The Geological Survey (USGS) (http://www.usgs.gov). These images were processed by visual interpretation with supervised classification supported by a geospatial analysis.In sub-basins S4 and S5, the base flow index (BFI) significantly increased, while runoff showed no significant trend. Comparing the results of land-use change, bare land was reduced in contrast with other sub-basins, and rangelands increased. It can be concluded that reducing rangelands and increasing vegetation caused a significant upward trend in these subbasins' baseflow index (BFI). Moreover, flood risk in these sub-basins increases due to the higher mean of precipitation and increasing base flow index (BFI) trend in sub-basins S1, S2, and S3. Due to a lower mean of precipitation, high development of farming land runoff reduced, and these areas led to dry and drought risk increase. This finding is in agreement with the results of a study conducted by Ashraf et al.^49^ indicated that in basin-scale groundwater depletions in Iran are mainly caused by extensive human water withdrawals. And the total groundwater depletion in Iran is estimated to be ~ 74 km^3^.

### Quantification of the impact of human interactions and elasticity estimation

The precipitation elasticity of runoff can calculate the influence of runoff on precipitation change without the influence of human activities (base period) and after abrupt change points. Based on the trend test and change points, the mean annual values, coefficient of variation (C_V_) of runoff, precipitation, and BFI index, and the elasticity coefficients during the base (before the change point) and impacted periods (after the change point) are listed in Table 7. In the subbasin S1 mean volume of runoff after the change point decreased − 1.49% compared with the base period but after the change point in the BFI index, runoff decreased − 55.02% while precipitation decreased − 2.31% which show the effect of the BFI index on runoff reduction that effected by human activities. Also in subbasin S2 that show after the change point, runoff decreased − 27.63% while precipitation decreased − 13.11% compared with the base period that shows the effect human activities had more significant effects on runoff reduction, in subbasin S3 that show after change point runoff decreased − 27.75% while precipitation decreased − 6.01% compared with the base period, in subbasin S4 mean volume of runoff after the change point decreased − 13.51% while precipitation decreased − 1.23% compared with the base period. In the subbasin S5 the situation is different; cause of human activities change point occurred in BFI index but no change point occurs in the runoff because the mountainous and snow-covered source of runoff in these sub-basins. On the other hand, during the period affected by human activities (according to the change point in the BFI index), the precipitation elasticity of runoff ${(\varepsilon }_{P})$ is lower than that in the period without the influence of human activities, which shows that runoff change with precipitation in sub-basins S1, S4, and S5 is resisted because of human activities, including land use and hydraulic engineering construction, in sub- basin S1 elasticity of runoff ${(\varepsilon }_{P})$ in the base period (without the influence of human activities) was 2.80 and after change point in BFIindex (the period affected by human activities) was − 0.65,also in subbasin S4 elasticity of runoff ${(\varepsilon }_{P})$ in the base period(without the influence of human activities) was 1.58 and after change point in BFIindex (the period affected by human activities) was − 0.46 and in sub-basin S5 elasticity of runoff ${(\varepsilon }_{P})$ in the base period(without the influence of human activities) was 2.79 and after change point in BFIindex (the period affected by human activities) was − 1.56. the situation in subbasin S2 are different; in the subbasin S2 cause of change point in precipitation elasticity of runoff ${(\varepsilon }_{P})$ is higher than the base period which that elasticity of runoff ${(\varepsilon }_{P}$) in the base period was 1.01 that is to say when the precipitation changes 10%, the runoff changes 10.1% but after change point elasticity of runoff ${(\varepsilon }_{P}$) was 1.90 it means the precipitation changes 10%, the runoff changes 19%.Consequently, runoff in this subbasin after change point become more sensitive to precipitation variation. In Table 7 change R refers to change point in the runoff, change B refers to change point in the baseflow index and change P refers to change point in the Precipitation.

**Table 7:** Calculation variation of precipitation, runoff, BFI index, and elasticity estimation.

Sub-basin	Period	Precipitation (mm)		Runoff (m^3^ s)		BF index		${\varepsilon }_{P}$
		Avg.	C_V_	Avg.	C_V_	Avg.	C_V_	
S1	Base period (1967–1976)	498.04	0.20	2.69	0.75	0.93	0.01	2.80
	Change R (1977–1993)	473.26	0.26	2.65	0.50	0.94	0.01	2.34
	Change (%)	− 4.98	0.06	− 1.49	− 0.25	0.01	0	− 0.46
	change B (1994–2016)	486.53	0.17	1.21	0.61	0.94	0.01	2.15
	Change (%)	− 2.31	− 0.03	− 55.02	− 0.14	0.01	0	− 0.65
S2	Base period (1967–1988)	553.15	0.22	0.76	0.89	0.95	0.01	1.01
	Change R& P (1989–2016)	480.62	0.25	0.55	1.19	0.95	0.01	1.90
	Change (%)	− 13.11	0.03	− 27.63	0.30	0	0	0.89
S3	Base period (1967–1988)	736.55	0.24	6.09	0.63	0.95	0.01	1.48
	Change R& B (1989–2016)	692.26	0.26	4.40	0.73	0.96	0.01	1.74
	Change (%)	− 6.01	0.02	− 27.75	0.10	0.01	0	0.26
S4	Base period (1967–1990)	961.38	0.27	1.85	0.56	0.91	0.02	1.58
	change B (1991–2016)	949.52	0.30	1.60	0.70	0.93	0.02	1.12
	Change (%)	− 1.23	0.03	− 13.51	0.14	0.02	0	− 0.46
S5	Base period (1967–1979)	789.06	0.14	15.30	0.44	0.95	0.01	2.79
	Change P (1980–1989)	765.85	0.29	16.30	0.30	0.95	0.01	0.54
	Change (%)	− 2.94	0.15	6.54	− 0.14	0	0	− 2.25
	change B (1990–2016)	735.70	0.29	16.86	0.52	0.96	0.01	1.23
	Change (%)	− 6.76	0.15	10.20	0.08	0.01	0	− 1.56

**Figure 8:**
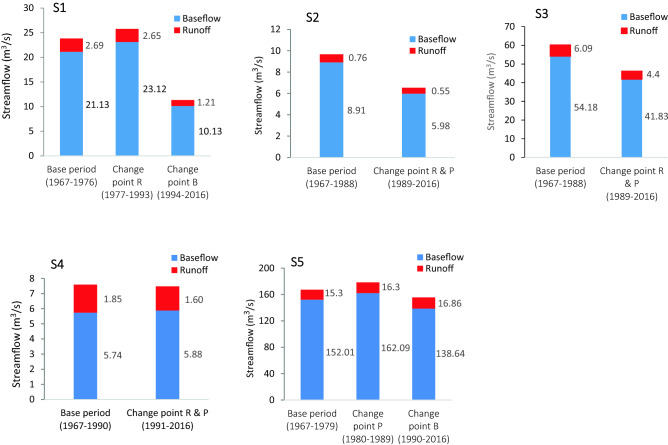
Baseflow and quick flow contributions in the streamflow after each change point in the P (precipitation), R (runoff), and B (BFI index) in the subbasins of the Dez River.

Figure 8 shows the contributions of components to the streamflow (baseflow and runoff) over the base period and after each change point year in every subbasin of the Dez River. As observed, the role of the baseflow to the streamflow reduced was significant. On the other hand, it expressed the groundwater depletions in these sub- basins. In the analysis land use change section also the role of the human activite was obvious.

## Conclusions

Changed the natural hydrological processes under combined influences of climate change and intensive human activities became major challenge for planning and management of water resources especially in the semi- arid area. In this study we have to assess the impact of climate change on the variation of streamflow using analysis precipitation data and assessing the impact of anthropogenic activities using an analysis baseflow index the result show in average precipitation at the upstream (subbasin S1, S2) is lower than downstream (sub-basins S4, S5) also, In the wet season (23 September to 21 June), maximum average precipitation occurs in the winter, fall, and spring season, respectively, in the dry season (22 June to 22 September) had a lowest rainy day with insignificant precipitation in all sub-basins of Dez river. Therefore, streamflow was relatively steady in the dry season, and the streamflow was nearly equal to baseflow. Fore analysis baseflow, Separation methods were analyzed and compared, and the suitable baseflow method for the Dez River basin was investigated. The comparison of the above results and the Hydrograph Separation Program (HYSEP) includes the fixed interval method (FIM) and Local minimum method (LMM). The fixed-interval method (FIM) and BFLOW method, including the Lyne and Hollick algorithm (LHA), underestimated the baseflow, while the sliding interval method (SIM) method was found to be effective for the separation of baseflow. Over the wet season, the contribution of baseflow to streamflow was also high. During the nonprecipitation period, the sliding interval method (SIM) produced baseflow similar to the streamflow, which agreed with the actual situation of the Dez River basin sub-basins. Based on the result of separation baseflow, the trend and abrupt points of precipitation, BFI index, and the M–K test determined runoff. The conclusions can be summarized as follows: (1) on the seasonal scale, precipitation significantly decreased only in the winter season, but runoff in winter and spring in sub-basins S1, S2, and S3 significantly decreased; moreover, on the annual precipitation scale, there was no significant trend, but runoff in sub-basins S1, S2, and S3 significantly decreased. The baseflow index (BFI) also significantly increased in sub-basins S1 and S3 in the winter and spring seasons and summer season and at the dry season annual scale in all sub-basins. The M–K test could detect the abrupt change points of precipitation, runoff, and the baseflow index (BFI).The abrupt change points of precipitation and runoff and the baseflow index (BFI) are different; therefore, the natural period considered before the change point and the affected period depends on the change point considered after the change point in the runoff, change point in the BFI index, and change point in precipitation. analysis and identification of change points in the long-term time series of the base period show the maximum change in subbasin S1 (subbasin of tireh river) gradual decrease after change point in runoff was − 1.49% but after change point in BFI index sudden occurrence of currency in runoff was − 55.2% in this subbasin precipitation was no significant. On the other hand, After change point from 1977 to 1993, comparison with the base period (from 1967 to 1976) elasticity estimation was − 0.46, but after change point in Baseflow index from 1994 to 2016 elasticity estimation was − 0.65. The baseflow index trend and elasticity estimation also indicated that intensive human activities had more significant effects on the Dez Basin's hydrological processes and streamflow variation. The results of this study show the occurrence of a flow decrease in the past and the possibility of a further decline in the future in the catchment area of Dez Dam. Given the importance of this basin in terms of hydropower production and water supply for drinking, industry, and agriculture, it is necessary to take steps to review the policy in the water sector of the basin (“Supplementary information S1”).

## Supplementary Information

Supplementary Information.
